# Canaloplasty for the treatment of primary open-angle glaucoma: A protocol of systematic review and meta-analysis

**DOI:** 10.1097/MD.0000000000020408

**Published:** 2020-05-29

**Authors:** Peng Sun, Hong-wei Liu, Ping-ping Zhou, Yan Meng

**Affiliations:** Department of Ophthalmology, First Affiliated Hospital of Jiamusi University, Jiamusi, China.

**Keywords:** Canaloplasty, efficacy, primary open-angle glaucoma, safety

## Abstract

**Background:**

Canaloplasty has been reported to manage primary open-angle glaucoma (POAG) effectively. However, no study has specifically and systematically investigated the efficacy and safety of canaloplasty for the treatment of POAG. Thus, this study will systematically and comprehensively appraise the efficacy and safety of canaloplasty for the treatment of POAG.

**Methods:**

MEDLINE, EMBASE, Cochrane Library, Web of Science, Cumulative Index to Nursing and Allied Health Literature, Allied and Complementary Medicine Database, Chinese Biomedical Literature Database, and China National Knowledge Infrastructure will be sought from the construction to the February 29, 2020. Only randomized controlled trials (RCTs) focusing on canaloplasty for the treatment of POAG will be included. Two reviewers will independently undertake selection of study, data extraction, and risk of bias assessment. Any doubts between 2 reviewers will be resolved through discussion with another experienced reviewer. RevMan 5.3 software will be employed for data analysis.

**Results:**

This study will summarize high-quality RCTs on investigating efficacy and safety of canaloplasty for the treatment of POAG.

**Conclusion:**

The findings of this study will help to determine whether canaloplasty is effective and safety for the treatment of POAG.

Systematic review registration: INPLASY202040119.

## 1 Introduction

Primary open-angle glaucoma (POAG) is the most common subtype of glaucoma, which is the leading cause of irreversible blindness.^[1–4]^ It is characterized by progressive degeneration of the optic nerve, and is associated with elevated intraocular pressure.^[5–7]^ It has been estimated that about 53 to 65 million people may affect POAG by 2020.^[8–10]^ Previous studies have reported that canaloplasty has been utilized for the treatment of POAG.^[11–21]^ However, the publication of the related systematic review and meta-analysis has not been identified in the databases. Thus, this study hopes to adopt systematic review to assess the efficacy and safety of canaloplasty for the treatment of POAG, and provide evidence for its application in clinical practice.

## 2 Methods and analysis

### 2.1 Study registration

We have registered this study on INPLASY202040119, and we have reported it according to the Preferred Reporting Items for Systematic Reviews and Meta-analysis Protocol.^[22]^

### 2.2 Study selection criteria

#### 2.2.1 Types of studies

Only randomized controlled trials (RCTs) of canaloplasty for the treatment of POAG will be included for inclusion. Non-RCTs, quasi-RCTs, case studies, and uncontrolled trials will be excluded.

#### 2.2.2 Types of participants

Patients who were diagnosed as POAG will be included, irrespective region, nation, sex, sex, and duration and severity of POAG.

#### 2.2.3 Types of interventions

As for experimental interventions, all patients received canaloplasty as their treatment.

As for comparators, participants were treated without restrictions related to their therapies, except canaloplasty.

#### 2.2.4 Types of outcomes

Primary outcomes include mean intraocular pressure, and change in best-corrected visual acuity.

Secondary outcomes consist of contrast sensitivity, bioelectric activity of the retina, rate of progression of glaucoma, blood flow to the eye and optic nerve head, quality of life, and incidence of adverse events.

### 2.3 Search strategy for study identification

#### 2.3.1 Electronic databases

The electronic databases (MEDLINE, EMBASE, Cochrane Library, Web of Science, Cumulative Index to Nursing and Allied Health Literature, Allied and Complementary Medicine Database, Chinese Biomedical Literature Database, and China National Knowledge Infrastructure) will be sought from the construction to the February 29, 2020. There are no limitations to the language and publication status. We will only consider randomized controlled trials (RCTs) for inclusion. The complete search strategy of Cochrane Library is created (Table 1). Similar search strategies will be built for other electronic databases.

**Table 1:**
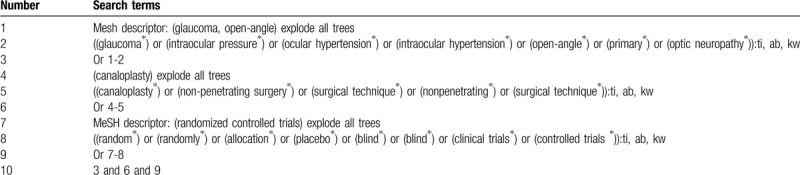
Search strategy of Cochrane Library.

#### 2.3.2 Other resources

We will examine other resources, such as dissertations, conference abstracts, clinical trial registry, and reference lists of included studies.

### 2.4 Study selection

Initial assessments will be carried out by 2 reviewers independently through filtrating the titles and abstracts of all searched citations. All duplicates and literatures that are clearly inconsistent with the study topic will be eliminated. After preliminary selection, full-text of potential studies will be carefully read against all inclusion criteria. When differences occur between 2 reviewers, we will invite another review to help reach a consensus by discussion. The results of literature selection will be shown in a flowchart.

### 2.5 Data extraction and management

Two reviewers will independently extract data from eligible articles by predesigned standard data extraction form. Any disagreements between 2 reviewers will be solved by another experienced reviewer through consultation. The form consists of title, first author, publication time, country, race, sex, age, sample size, eligibility criteria, diagnostic criteria, trial design, intervention details, control specifics, outcomes, safety, and, other related information.

### 2.6 Missing data dealing with

In the event of data loss during the period of study selection and data extraction, we will contact primary research authors by email, telephone to request the lost data. If the lost data cannot be obtained, we will analyze the available data only.

### 2.7 Risk of bias assessment

Two reviewers will independently evaluate the study quality for each article using Cochrane Collaboration's bias risk assessment tool.^[23]^ Each article will be assessed through 7 domains, and each one is further divided into low, unclear, and high risk of bias. Any confusion will be answered by consensus with another experienced reviewer.

### 2.8 Grading quality of evidence

Two reviewers will appraise quality of evidence by Grading of Recommendations Assessment, Development, and Evaluation tool.^[24]^ Disagreements between 2 reviewers will be settled by another experienced reviewer through discussion.

### 2.9 Statistical analysis

#### 2.9.1 Data synthesis

Data analysis will be performed using RevMan 5.3 software. As for continuous data, we estimate it as weighted mean difference or standardized mean difference and 95% confidence intervals (CIs). As for dichotomous data, we will express it as risk ratio and 95% CIs. Heterogeneity across articles will be detected using *χ*^2^ test and *I*^2^ test. When *P* ≥0.1 or/and *I*^2^ ≤50%, minor heterogeneity is considered, and a fixed-effect model will be used. Otherwise, when *P* < 0.1 or/and *I*^2^ > 50%, obvious heterogeneity is regarded, and a random-effect model will be utilized. If possible, we will carry out a meta-analysis based on the similar characteristics of study and patient, types of interventions and controls, and outcomes. If considerable heterogeneity is found, we will perform subgroup analysis and sensitivity analysis to explore possible sources of such heterogeneity.

#### 2.9.2 Subgroup analysis

If necessary, a subgroup analysis will be carried out according to the different study characteristics, study quality, treatments, and controls.

#### 2.9.3 Sensitivity analysis

We will employ a sensitivity analysis to test the robustness of results by removing low quality of studies.

#### 2.9.4 Reporting bias

We will conduct a funnel plot and Egger regression analysis to explore reporting bias when >10 articles are included.

### 2.10 Dissemination and ethics

We will plan to publish this study through a peer-reviewed journal. This study will not collect individual patient data; thus, ethical approval is unnecessary for this study.

## 3 Discussion

At present, a variety of studies have reported the efficacy and safety of canaloplasty for the treatment of POAG.^[11–21]^ However, as far as we know, no literature review systematically assesses the efficacy and safety of canaloplasty for POAG. Therefore, this systematic review will investigate the efficacy and safety of POAG by canaloplasty treatment. We expect that this study may provide a basis for canaloplasty for the treatment of POAG, and may provide better options for the treatments to such patients.

## Author contributions

**Conceptualization:** Peng Sun, Hong-wei Liu, Ping-ping Zhou, Yan Meng.

**Data curation:** Peng Sun, Hong-wei Liu, Yan Meng.

**Formal analysis:** Hong-wei Liu, Ping-ping Zhou.

**Funding acquisition:** Yan Meng.

**Investigation:** Yan Meng.

**Methodology:** Peng Sun, Hong-wei Liu, Ping-ping Zhou.

**Project administration:** Yan Meng.

**Resources:** Hong-wei Liu, Ping-ping Zhou.

**Software:** Peng Sun, Hong-wei Liu, Ping-ping Zhou.

**Supervision:** Yan Meng.

**Validation:** Peng Sun, Hong-wei Liu, Yan Meng.

**Visualization:** Peng Sun, Ping-ping Zhou, Yan Meng.

**Writing – original draft:** Peng Sun, Hong-wei Liu, Ping-ping Zhou, Yan Meng.

**Writing – review & editing:** Peng Sun, Ping-ping Zhou, Yan Meng.
